# Supplementary material for: Global and Sex-Stratified Genome-Wide Association Study of Long COVID Based on Patient-Driven Symptom Recall
Source: Int J Mol Sci. 2025 Sep 22;26(18):9252. doi: 10.3390/ijms26189252 (PMC12471052; doi:10.3390/ijms26189252)
Supplement: Supplementary file 1 [file ijms-26-09252-s001.zip › ijms-3829756-supplementary.pdf]

Supplementary Material for

Sex-specificity and functional annotation of genetic characteristics associated to long COVID in a genome-wide association study

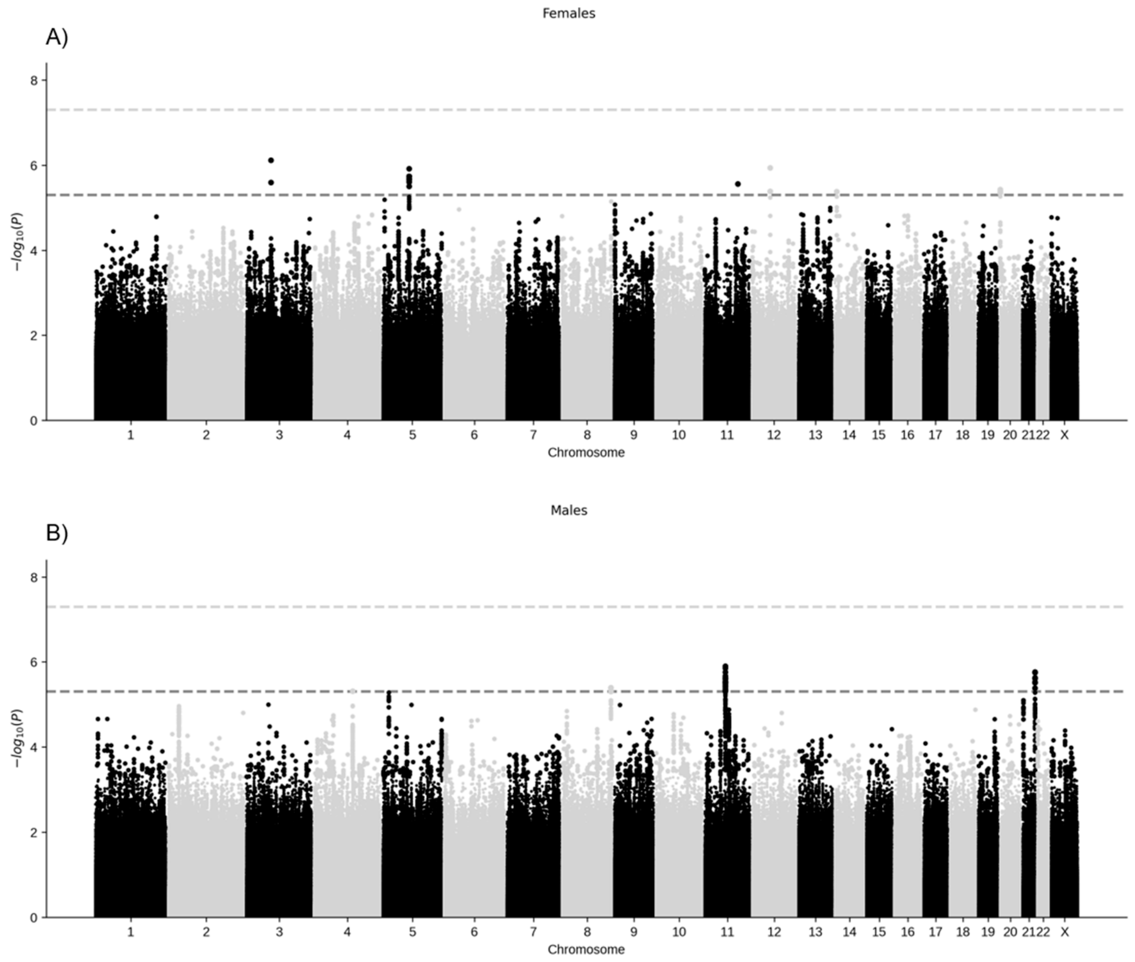

Supplementary Figure S1. Long COVID Manhattan plots for the sex-stratified (A: females; B: males), sensitivity analyses. Dark grey and light grey lines are the *p*-value significance thresholds $5x10^{-6}$ and $5x10^{-8}$, respectively.

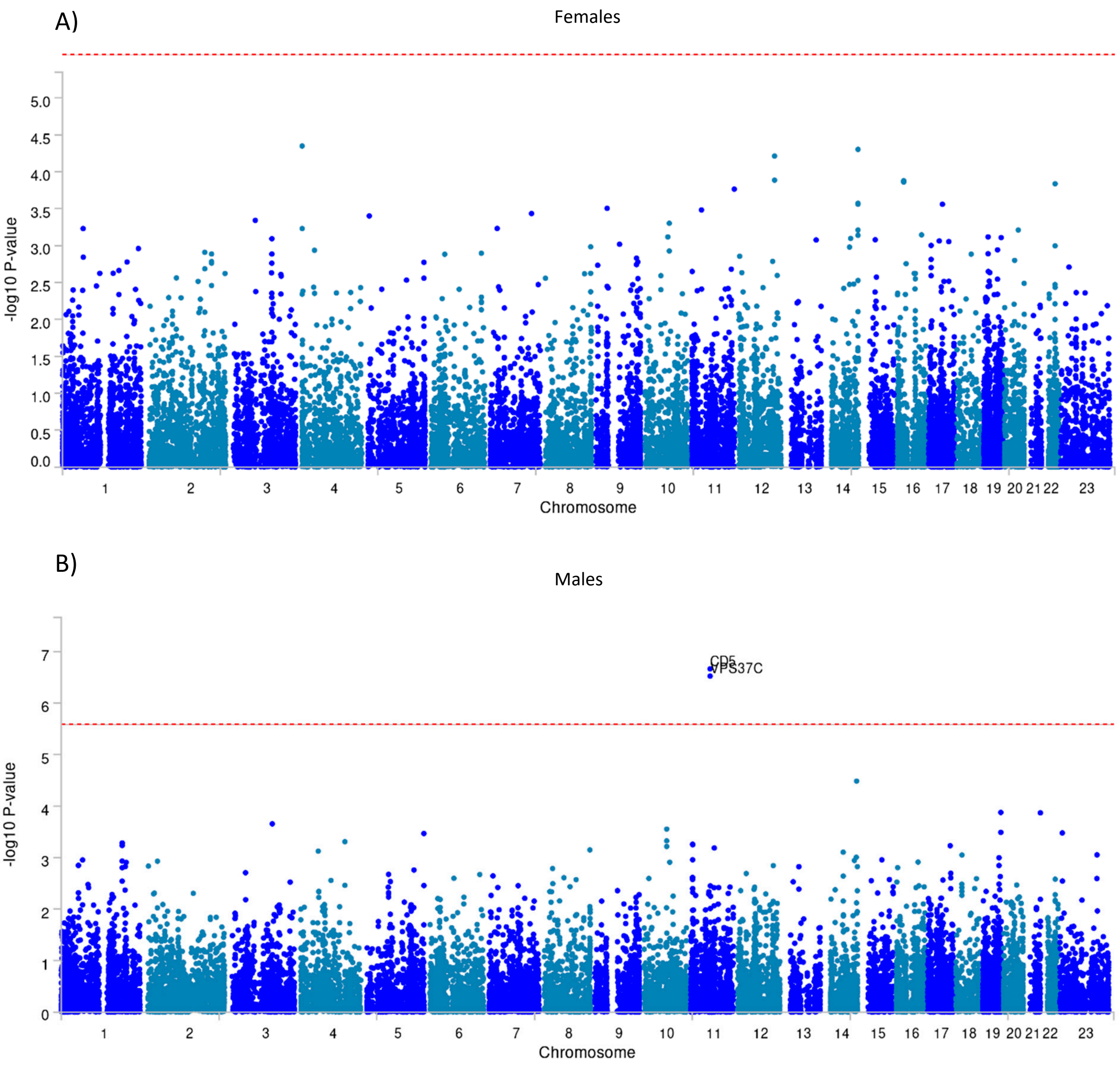

Supplementary Figure S2. MAGMA gene-based analysis in A) females and B) males. The red line represents the p-value significance threshold, which corresponds to $0.05/19,394 = 2.578x10^{-6}$

Supplementary Table S1. Genomic risk loci of the multi-ancestry and European sensitivity analyses in males.

**Multi-ancestry study (males)**					
**Genomic risk locus**	Chromosome	BP start (GRCh38)	BP end (GRCh38)	Lead SNP	uniqID (GRCh38)
**1**	1	38970826	38989754	rs10888603	1:38972945:A:C
**2**	4	111393685	111595181	rs2717199	4:111595181:A:G
**3**	11	61086514	61192664	rs2186409	11:61127020:G:T
**4**	19	50468235	50507099	rs1274686	19:50495701:C:T
**5**	21	46339323	46544730	rs11700596	21:46454141:C:G
**European sensitivity study (males)**					
**Genomic risk locus**	Chromosome	BP start (GRCh38)	BP end (GRCh38)	Lead SNP	uniqID (GRCh38)
**1**	4	111393684	111595181	rs2717199	4:111595181:A:G
**2**	8	141529615	141563374	rs13268039	8:141537433:C:G
**3**	11	61093907	61192328	rs7125216	11:61160331:C:T
**4**	21	46301313	46544730	rs56134005	21:46468232:C:T
